# Supplementary material for: Linker histone H1 prevents R-loop accumulation and genome instability in heterochromatin
Source: Nat Commun. 2017 Aug 18;8:283. doi: 10.1038/s41467-017-00338-5 (PMC5561251; doi:10.1038/s41467-017-00338-5)
Supplement: Supplementary file 2 — Supplementary Information [file 41467_2017_338_MOESM2_ESM.pdf]

Title: Supplementary Information

Description: Supplementary Figures and Supplementary Tables

Title: Peer Review File

Description:

Supplementary Table 1. dH1 interacting factors with activities that could regulate R-loops dynamics

RNA helicases	DNA helicases	DNA topoisomerases	RNA transport	RNA processing
Upf1	rept	scf	thoc5	asf1
Su(var)2-10	Psf3	pho	PCID2	U2af50
l(2)35Df	pont		CG5857	NHP2
Dbp21E2	Mcm5		CG5382	dbe
Hel25E	Mcm7		CG4572	hnRNP36
Dbp73D			CG5053	hnRNP48
CG9630			CG8671	CG11266
CG7878				CG16941
CG6227				CG17540
CG4901				CG5454
CG3561				CG6227
				CG6995
				CG10909
				CG12301
				CG1542
				CG6712

Supplementary Table 2. Primers used in ChIP-qPCR, DRIP-qPCR and RT-qPCR experiments

**Mthl12-F**	TGCAAAGCTATTTAATGCACGA
**Mthl12-R**	TAAAACTCGACTGGGGTTCC
**Diver2LTR-F(1)**	CGCCAAAACTGTGCAGTAGA
**Diver2LTR-R(1)**	CAGATAAATGCGTGCGAGAA
**Diver2ORF-F**	CTCTTACGGTGAGGCTGGAG
**Diver2ORF-R**	GCTGTCGATTATGCTCGTGA
**GATELTR-F(1)**	CCGCTCTTCACCTCAGAGTC
**GATELTR-R(1)**	CCGGGCGTATGTTTATTCAG
**GATEORF-F**	GCAGACCTGGCAAGTAGAGG
**GATEORF-R**	GAGCGTTGACCTGAGTAGGC
**Invader4LTR-F(1)**	AGATGACAATGTGGCACACG
**Invader4LTR-R(1)**	GATCGACGTCAGCAGTCAAA
**Invader4ORF-F**	GCTTACGCCTTCAAGAAACG
**Invader4ORF-R**	CAAAAATGGCACATGGTCTG
**PogoIR-F(1)**	CATCGGCAAGATATCTGCATT
**PogoIR-R(1)**	CGATGCAGCAAACGTATGAA
**PogoORF-F**	TACATTTGGTTCGGACAGCA
**PogoORF-R**	ACGTGCCGGTCAAGAATTAC
**3S18LTR-F(1)**	CAGCGGAATCAATGTAAGCA
**3S18LTR-R(1)**	TGGAAAAGTACTGGGCAAGC
**3S18ORF-F**	GCCATCAATCGCTTCTTCTC
**3S18ORF-R**	CCAGGAAAGCTTCGTACTGC
**Het-AUTR-F(1)**	TTCGCTTGCCAAAGACTCTC
**Het-AUTR-R(1)**	GCTTTTCTTTGCAGCCTGAG
**Het-AORF-F**	AAACGACGATCTGGACTGCT
**Het-AORF-R**	CGGAAAAATGCTGGGAGTTA
**GYPSYLTR-F(1)**	GGCTCATTGCCGTTAAACAT
**GYPSYLTR-R(1)**	GGCGATAGCGATTTGATTGT
**GYPSYORF-F**	CCTCAGAGCTGTGGTCTTCC
**GYPSYORF-R**	CAGATGGCAGGTCTTTTGGT
**DOCUTR-F(1)**	GACATTCGGCATTCCACAGT
**DOCUTR-R(1)**	ACGTCTCCACCCGAAGACT
**DOCORF-F**	CGCTGTGCCAGCTGTAAATA
**DOCORF-R**	ATTGTTGTTGCAAACGGTCA
**MDG3LTR-F(1)**	TCAGTCGCTGTTGAACCAAG
**MDG3LTR-R(1)**	TTAGCCGCCGTTTACAGAAG
**MDG3ORF-F**	AAATGCAAAAAGGCCAAATG
**MDG3ORF-R**	AGCTAAACGGTTTCGGGTTT
**ACCORDLTR-F(1)**	TAGGCGACATCAGCAAAGTG
**ACCORDLTR-R(1)**	ATCGGGTGCAACAGAGTTTC
**ACCORDORF-F**	CCAACAGCAACAACATGGAC
**ACCORDORF-R**	AAAAGCCAAAATGTCGGTTG
**DMCR1AUTR-F(1)**	TTCGCGAGTGATTCTTTGTG
**DMCR1AUTR-R(1)**	ACAACGCACAAAAGGGAGAG
**DMCR1AORF-F**	GTTGTGATGCTTGCCTTGTG

**DMCR1AORF-R**	ATTTCATCTCGTTCGCAACC
**TARTORF-F**	CCAATGCAACCAAAGCATTA
**TARTORF-R**	TATGTGTGGGAGGGAGAAGC
**TARTUTR-F**[(1)]	GACTTCCACTCCCTGCAGAC
**TARTUTR-R**[(1)]	TCCTTCACATCCAGGGAATC
rDNANTS1-F	GAAAAGCCATTTTAGTGAATGGA
rDNANTS1-R	CGTCGTAGAACAGCTAGCTTACAG
rDNANTS2-F	GGTTGCCGACCTCTCATATT
rDNANTS2-R	TGCCAAAATCATATGAACACA
**Sat.DNA-F**	AAACACGTCTCCACCCGAAG
**Sat.DNA-R**	CTATTCTAACATTCGGCATTCCAC
**RpL5-F**	CGGTATGAACGATATGACAGG
**RpL5-R**	TCACATGGCTATTGAAGTGAAA
**CG9662-F**	CCCTGCATTGGTGTATTTCC
**CG9662-R**	TGACATGGCTTACGATGCAG
**CG125672-F**	ACCGATTGCTTTTCGTTGAA
**CG125672-R**	GCTTCGATGCTTATTTTATATCCAG
**T7dH1-F**	ATTGTAATACGACTCACTATAGATGTCTGATTCTGCA GTTGC
**T7dH1-R**	ATTGTAATACGACTCACTATAGTTACTTTTTGGCAGC CGTAG
**T7HP1a-F**	TAATACGACTCACTATAGGGAGAATCCCGAAACTGAG AACACG
**T7HP1a-R**	TAATACGACTCACTATAGGGAGATCCGATGCCTTAAG AGTTGG
**LacZ-F**	TAATACGACTCACTATAGGGATGACCATGATTACGCC AAGC
**LacZ-R**	TAATACGACTCACTATAGGGCAATTTCCATTCGCCAT TCAG
**Tub-F**	ACCTGAACCGTCTGATTGGC
**Tub-R**	GCAGAGAGGCGGTAATCGAG
**Act-F**	CGTCCACCATGAAGATTAAGATTGT
**Act-R**	CAATACTTTTGACTCCCATCCTTTG
**RNH1-F**	ATGAGCTGGCTTCTGTTCC
**RNH1-R**	TCAGTCTTCCGATTGTTTAGC

[(1)] not used for RT-qPCR

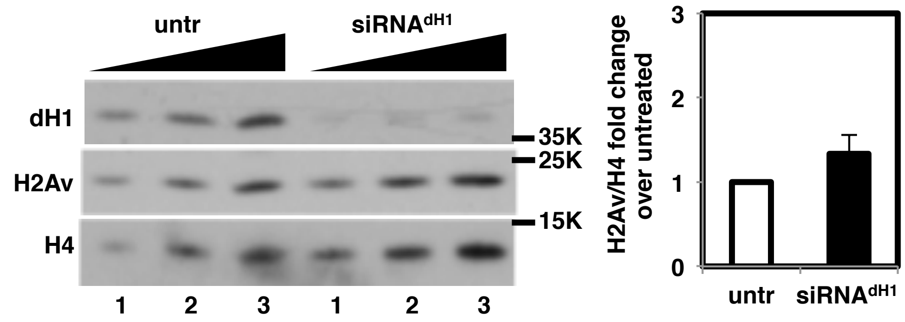

Supplementary Figure 1. dH1 depletion does not affect H2Av content. WB analyses with αdH1, αH2Av and αH4 antibodies of increasing amounts of extracts (lanes 1-3) prepared from $siRNA^{dH1}$ and untreated cells. The positions corresponding to molecular weight markers are indicated. On the right, quantitative analysis of the results (N= 3). Error bars are s.e.m. The observed difference is not statistically significant (p-value= 0.171; two-tailed Student's t-test).

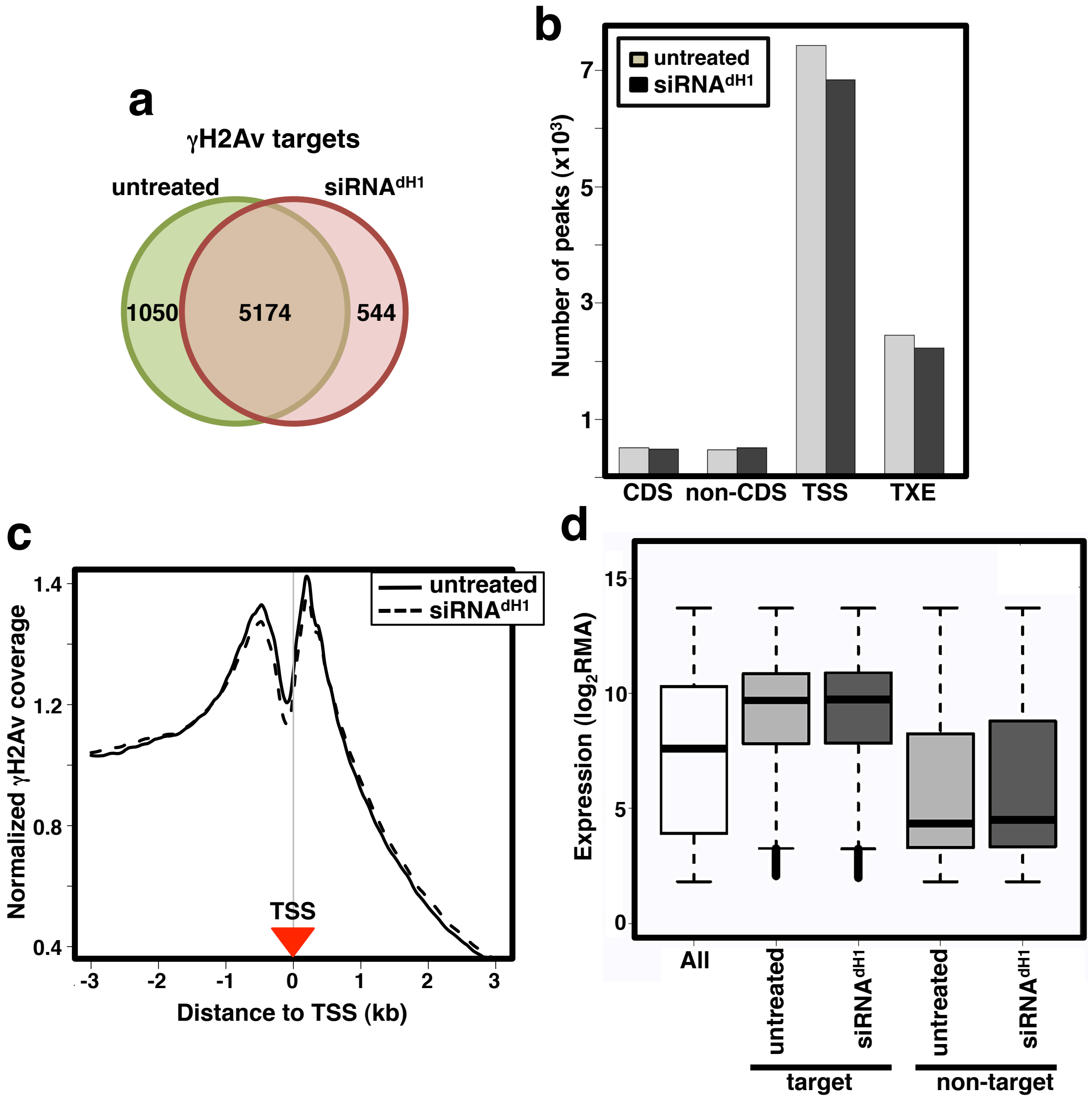

Supplementary Figure 2. γH2Av ChIP-seq analysis. a) Venn diagram showing the intersection between γH2Av targets in control untreated and dH1-depleted cells. **b**) The number of γH2Av peaks mapping to coding regions (CDS), non-coding regions (non-CDS), transcriptional start sites (TSS) or transcriptional ends (TXE) is presented for control untreated and dH1-depleted cells. **c**) γH2Av distribution around TSS is presented for control untreated and dH1-depleted cells. For each gene, the coverage profile was normalized dividing by the average coverage in that gene. The position of TSS is indicated. **d**) Box plot showing the expression in S2 cells of γH2Av target and non-target genes identified in untreated and dH1-depleted cells.

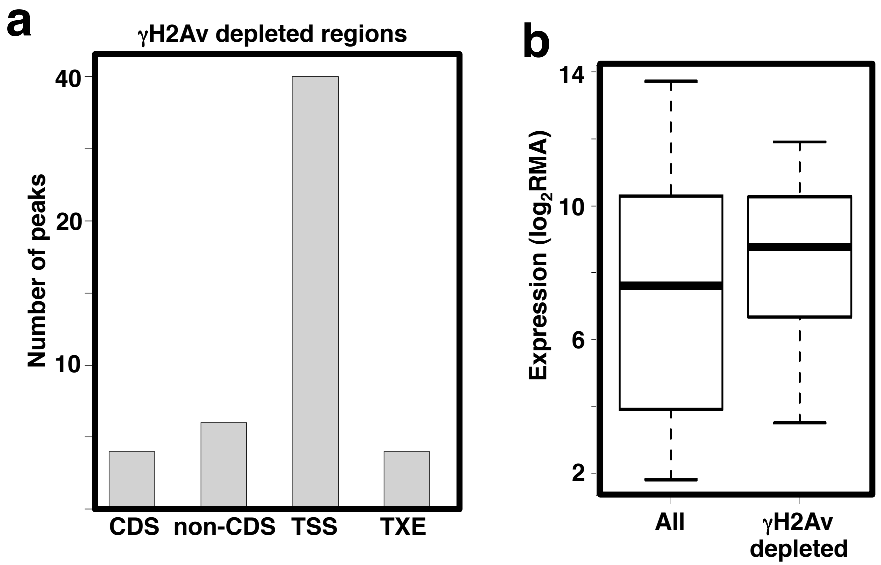

Supplementary Figure 3. Analysis of regions where γH2Av specifically decreases upon dH1 depletion . a) The number of peaks showing specific γH2Av depletion in dH1-depleted cells that map to coding regions (CDS), non-coding regions (non-CDS), transcriptional start sites (TSS) or transcriptional ends (TXE) is presented. **b**) Box plot showing the expression in S2 cells of genes showing specific γH2Av depletion in dH1-depleted in comparison to that of all genes.

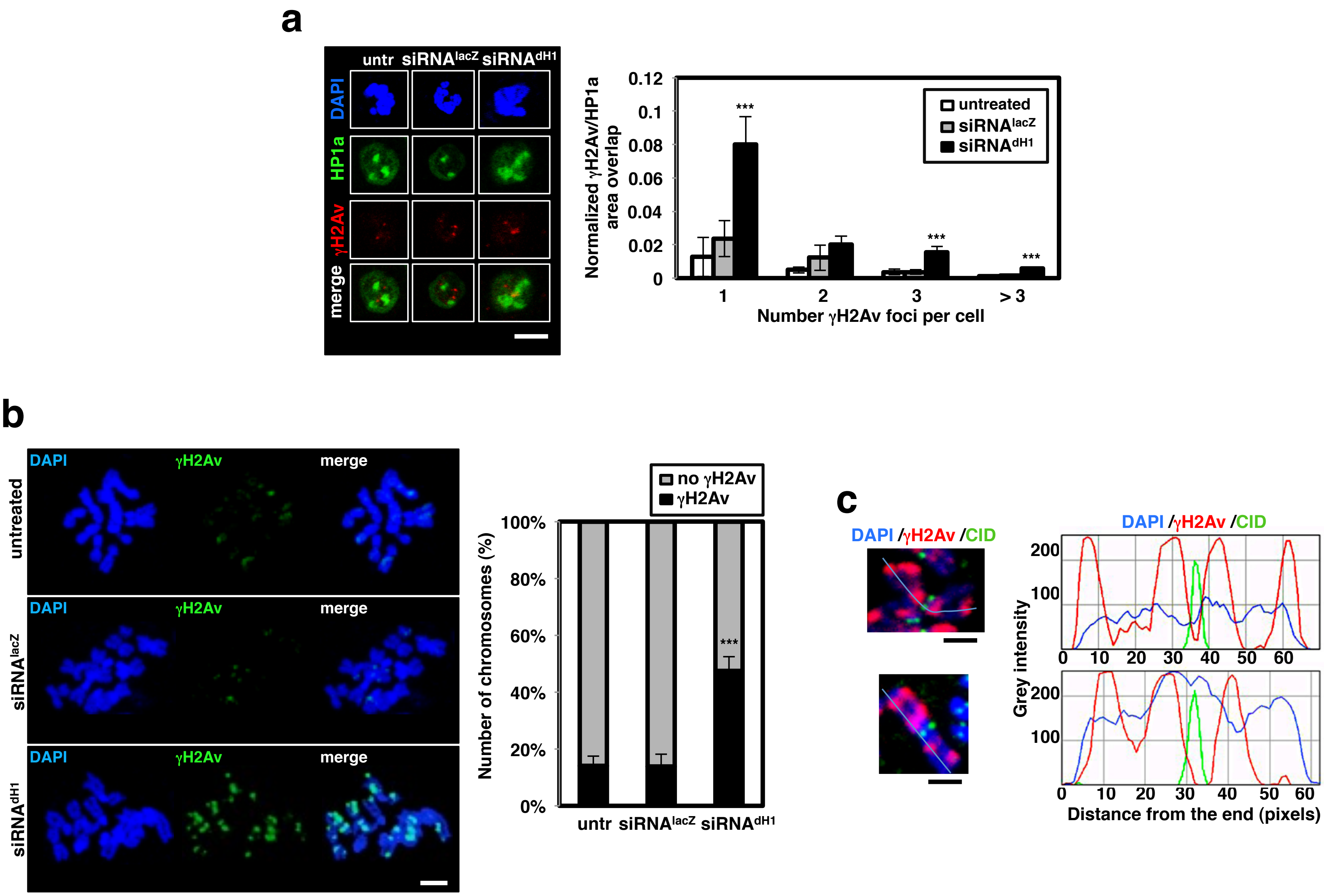

Supplementary Fig 4

Supplementary Figure 4. αγH2Av immunostainings of dH1-depleted cells and metaphase chromosomes. a) Immunostainings with αγH2Av (red) and αHP1a (green) in $siRNA^{dH1}$, $siRNA^{lacZ}$ and untreated cells. DNA was stained with DAPI (blue). Scale bar corresponds to 4μm. On the right, the extent of γH2Av/HP1a colocalization determined as the proportion of total γH2Av area overlapping with αHP1a signals normalized respect to the total αHP1a area is presented as a function of the number of γH2Av foci per cell (n>50 for each condition). Error bars are s.e.m. The p-values of $siRNA^{dH1}$ respect to $siRNA^{lacZ}$ are indicated (no asterisk >0.05, ***<0.005; two-tailed Student's t-test). **b**) Immunostainings with αγH2Av antibodies (green) of metaphase chromosomes from $siRNA^{dH1}$, $siRNA^{lacZ}$ and untreated cells. DNA was stained with DAPI (blue). Scale bar corresponds to 4μm. On the right, the relative percentage of metaphase chromosomes from $siRNA^{dH1}$, $siRNA^{lacZ}$ and untreated cells showing αγH2Av reactivity or not is presented (n>100 for each condition). Error bars are s.e.m. The p-values of $siRNA^{dH1}$ respect to $siRNA^{lacZ}$ are indicated (***<0.005; two-tailed Student's t-test). **c**) Immunostainings with αγH2Av (red) and αCID (to label centromeres (green)) of $siRNA^{dH1}$ metaphase chromosomes. DNA was stained with DAPI (blue). Scale bars are 2μm. On the right, grey intensity profiles along the indicated lines on the chromosomes (in light blue) are presented from left to right.

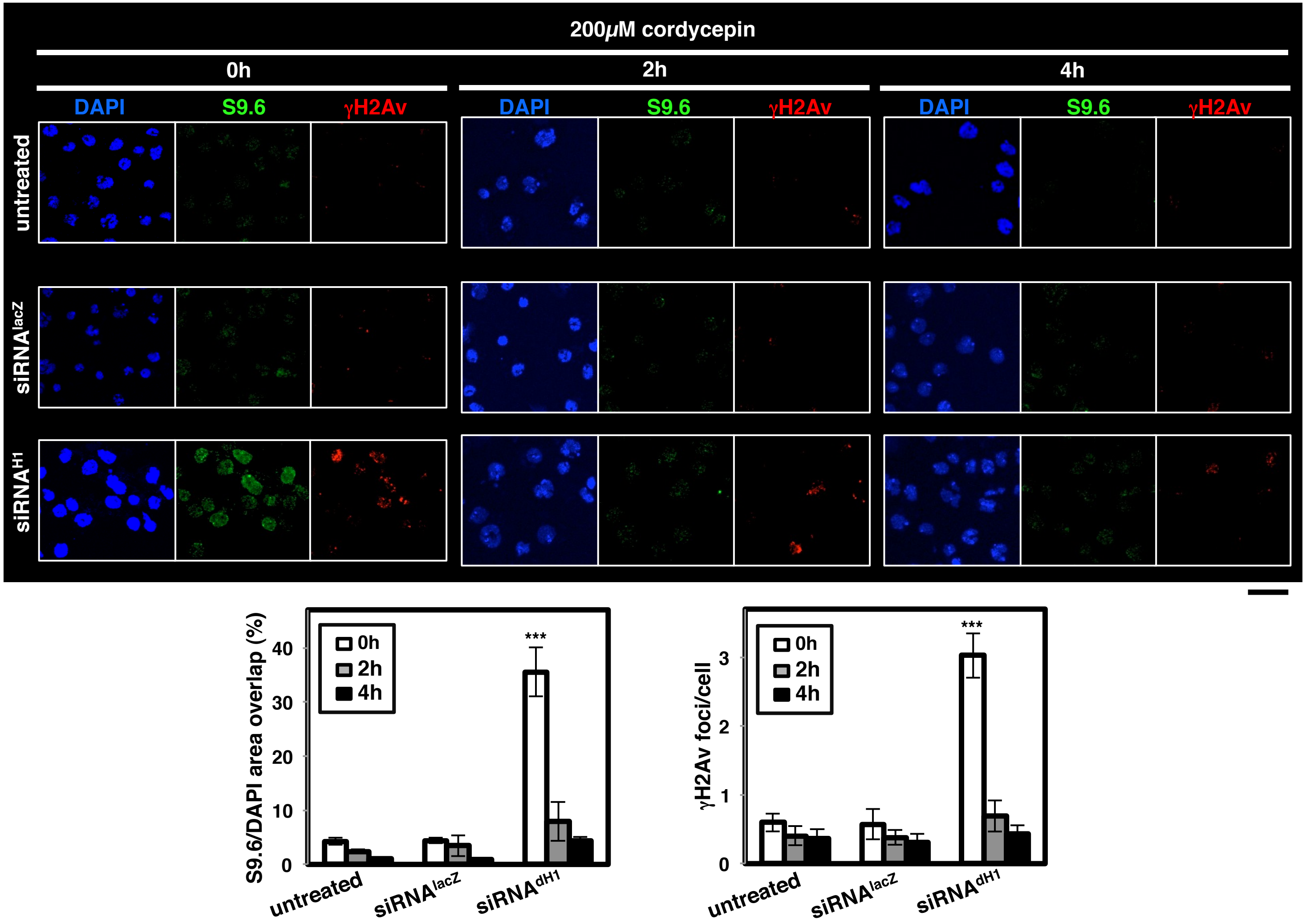

Supplementary Figure 5. Cordycepin treatment abolishes R-loops accumulation induced by dH1 depletion. Immunostainings with S9.6 (green) and αγH2Av (red) antibodies of $siRNA^{dH1}$, $siRNA^{lacZ}$ and untreated cells after incubation with 200μM cordycepin for 0h, 2h and 4h. DNA was stained with DAPI (blue). Scale bar corresponds to 15μm. On the bottom, S9.6 (left) and γH2Av (right) reactivities are determined as the proportion of DAPI area stained with S9.6 antibodies and the number of γH2Av foci per cell, respectively (n> 50 for each condition). Error bars are s.e.m. The p-values of $siRNA^{dH1}$ respect to $siRNA^{lacZ}$ are indicated (no asterisk>0.05, ***<0.005; two-tailed Student's t-test).

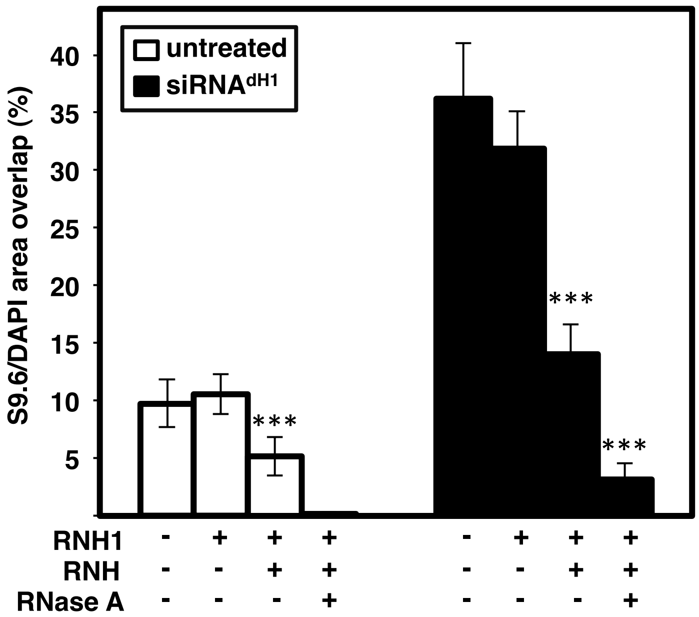

Supplementary Figure 6. **S9.6 reactivity of S-phase sorted cells.** S9.6 reactivity of siRNA^{dH1} and untreated cells sorted at S-phase overexpressing human RNH1 and treated *in vitro* with bacterial RNH and RNAse A as indicated is presented as the proportion of DAPI area stained with S9.6 antibodies (n> 50 for each condition). Error bars are s.e.m. The p-values respect to cells not expressing RNH1 and not treated *in vitro* with bacterial RNH and RNAse A are indicated (no asterisk> 0.05, ***< 0.005; two-tailed Student's t-test).

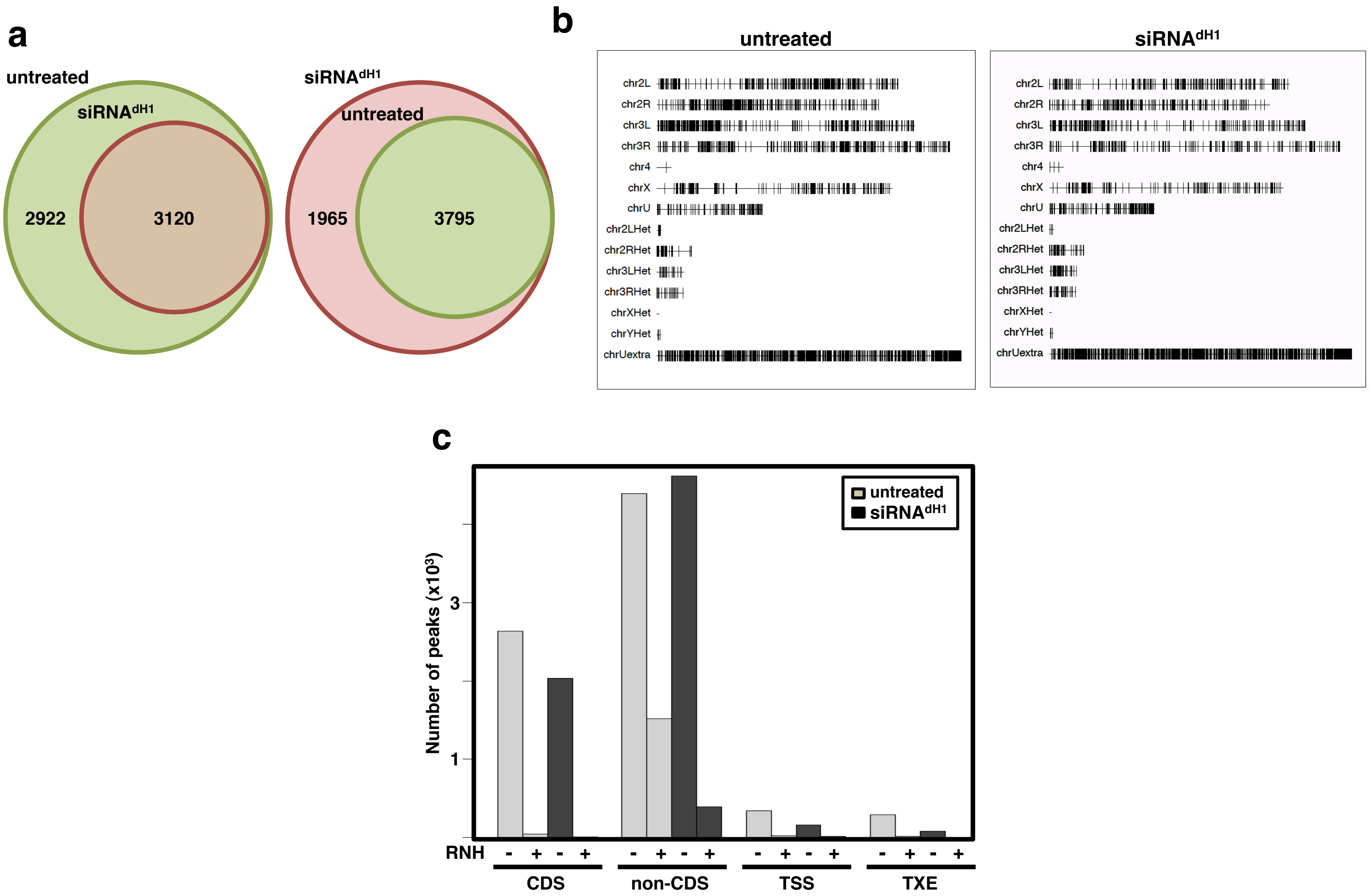

Supplementary Fig 7

Supplementary Figure 7. DRIP-seq analysis. a) Euler diagram showing the overlap of S9.6 peaks detected in control untreated cells with those detected in dH1-depleted cells (left) and *vice versa* (right). **b**) Chromosomal distribution of S9.6 peaks detected in control untreated (left) and dH1-depleted cells (right). 2L and 2R, and 3L and 3R correspond to chromosome 2 and 3 left and right arms respect to the position of the centromere, respectively. Chromosome 4 and X are oriented with the centromere to the right. 2LHet, 2RHet, 3LHet, 3RHet, XHet and YHet correspond to partially assembled pericentromeric heterochromatin regions of the indicated chromosomes. U and Uextra correspond to unassembled highly repetitive chromosome regions. **c**) The number of S9.6 peaks mapping to coding regions (CDS), non-coding regions (non-CDS), transcriptional start sites (TSS) or transcriptional ends (TXE) is presented for control untreated and dH1-depleted cells treated (+) or not (-) with bacterial RNase H (RNH).

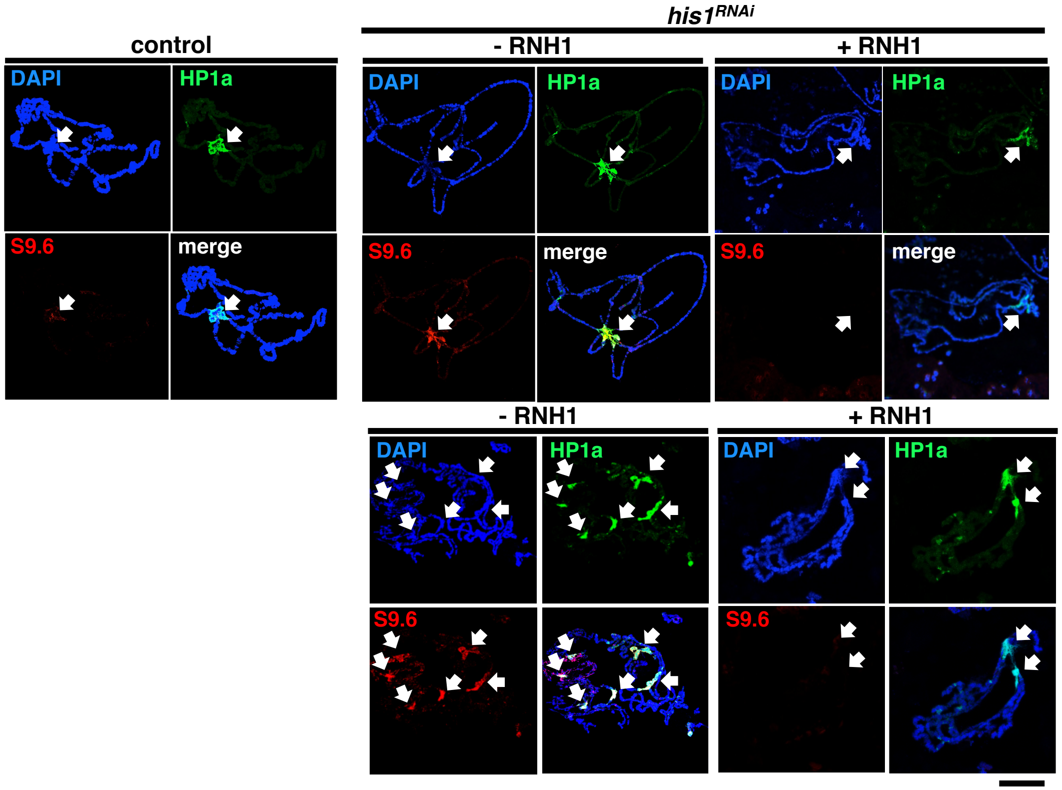

Supplementary Figure 8. R-loops induced by dH1 depletion accumulate in heterochromatin. Immunostainings with S9.6 (red) and αHP1a (green) of polytene chromosomes from dH1-depleted *his1*^{RNAi} expressing human RNH1 (+) or not (-), and from control *GFP*^{RNAi} flies. Arrows indicate the heterochromatic chromocenter. dH1-depleted *his1*^{RNAi} chromosomes showing chromocenters disorganized into several HP1a enriched regions are shown on the bottom. DNA was stained with DAPI (blue). Scale bar corresponds to 30μm.

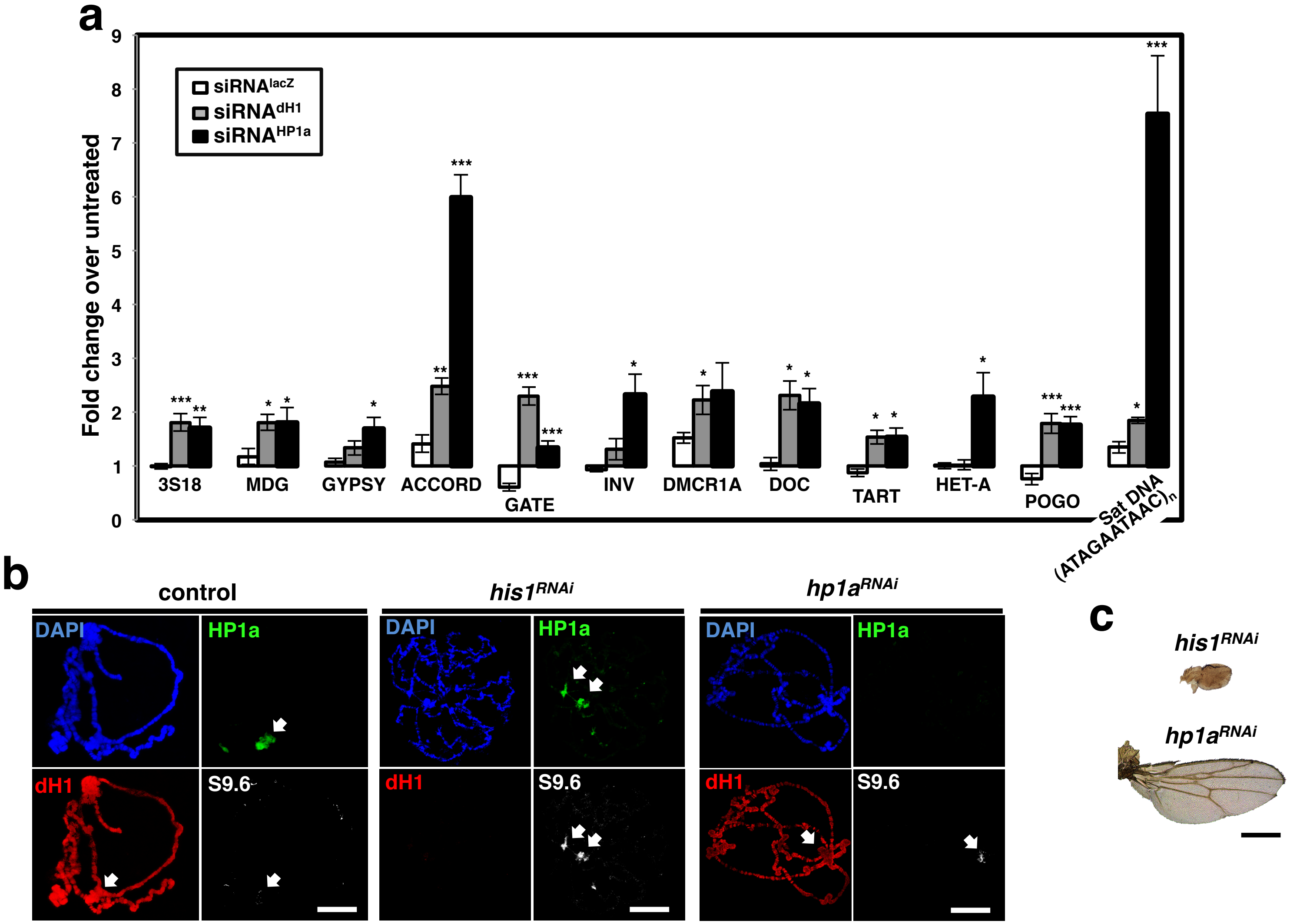

Supplementary Figure 9. HP1a depletion does not induce R-loops accumulation. a) RT-qPCR analyses of the indicated repetitive elements in siRNA^{dH1}, siRNA^{HP1a} and siRNA^{lacZ}. The fold change of expression respect to untreated cells is plotted (N= 2). Error bars are s.e.m. The p-values of siRNA^{dH1} and siRNA^{HP1a} respect to siRNA^{lacZ} are indicated (no asterisk>0.05, *< 0.05, **<0.01, ***<0.005; two-tailed Student's t-test). **b**) Immunostainings with αHP1a (green), αdH1 (red) and S9.6 (white) antibodies of polytene chromosomes from control undepleted flies (left), dH1-depleted *his1*^{RNAi} flies (centre) and HP1a-depleted *hp1a*^{RNAi} flies (right). Arrows indicate the chromocenter. DNA was stained with DAPI (blue). Scale bar corresponds to 30μm. **c**) Wings from dH1-depleted *his1*^{RNAi} flies (top) and HP1a-depleted *hp1a*^{RNAi} flies (bottom). Scale bar corresponds to 500μm.

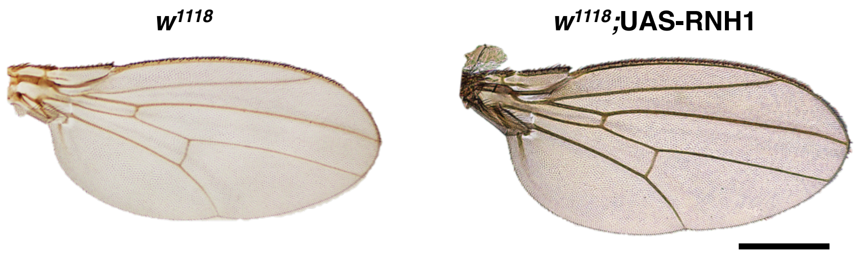

Supplementary Figure 10. RNH1 overexpression in the wing imaginal disc does not affect wing development. Wings from w^{1118} flies overexpressing human RNH1 in the pouch region of the wing imaginal disc (right) or not (left). Scale bar corresponds to 500μm.

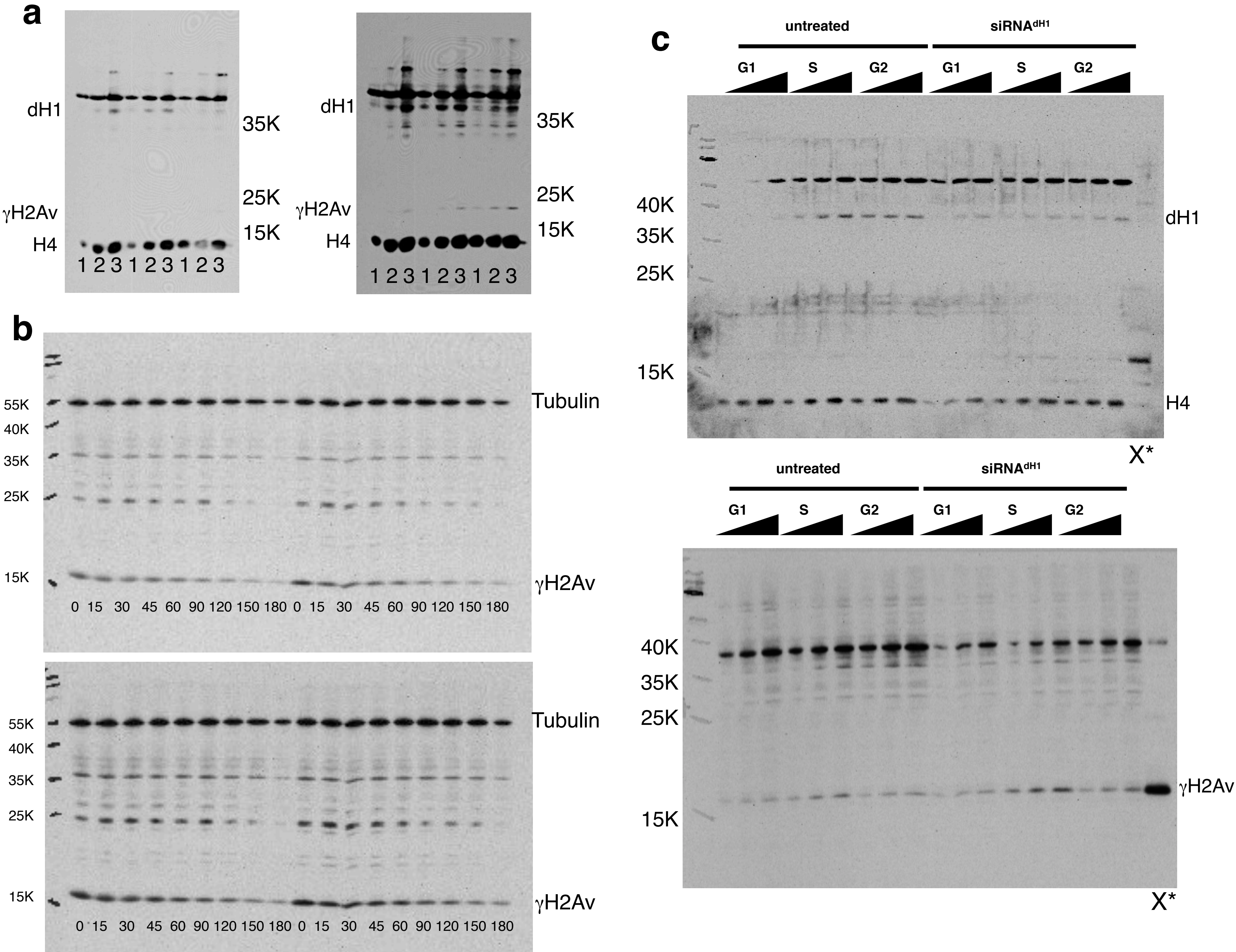
a
dH1
35K
25K
γH2Av
15K
H4
1 2 3 1 2 3 1 2 3
dH1
35K
25K
γH2Av
15K
H4
1 2 3 1 2 3 1 2 3
b
55K
40K
35K
25K
15K
Tubulin
γH2Av
0 15 30 45 60 90 120 150 180 0 15 30 45 60 90 120 150 180
55K
40K
35K
25K
15K
Tubulin
γH2Av
0 15 30 45 60 90 120 150 180 0 15 30 45 60 90 120 150 180
c
untreated
siRNA dH1
G1
S
G2
G1
S
G2
40K
35K
25K
15K
dH1
H4
X*
untreated
siRNA dH1
G1
S
G2
G1
S
G2
40K
35K
25K
15K
γH2Av
X*

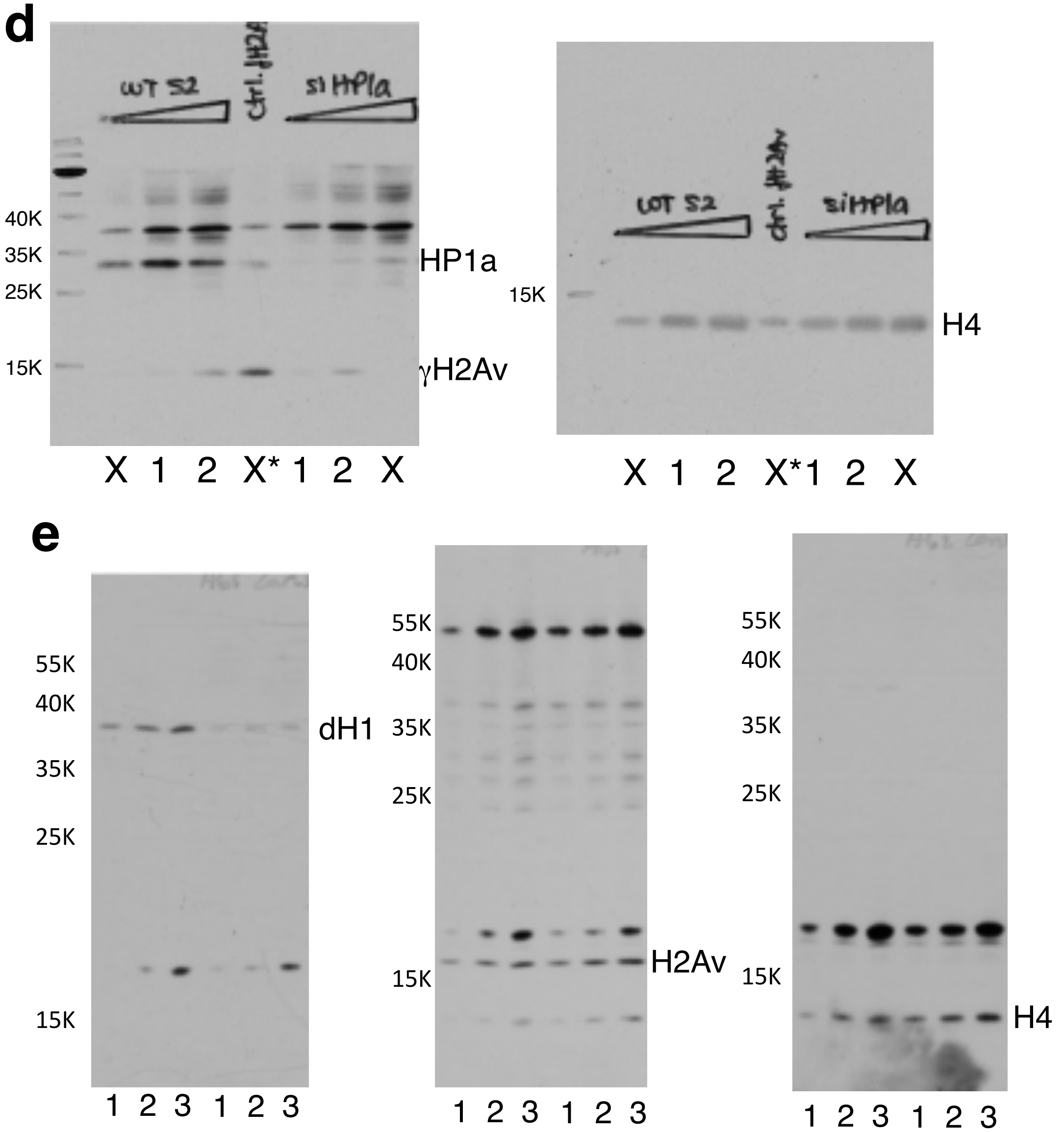

Supplementary Figure 11. Uncropped scans of the WBs shown in: (a) Fig. 1b (two different exposures are presented); (b) Fig. 1d (two different exposures are presented); (c) Fig. 4d (lane labeled X* corresponds to a control of irradiated cells); (d) Fig. 7b (lane labeled X* corresponds to a control of irradiated cells), and (e) Supplementary Fig. 1.
